# Genital self-sampling compared with cervicovaginal lavage for the diagnosis of female genital schistosomiasis in Zambian women: The BILHIV study

**DOI:** 10.1371/journal.pntd.0008337

**Published:** 2020-07-14

**Authors:** Amy S. Sturt, Emily L. Webb, Comfort R. Phiri, Tobias Mweene, Namakau Chola, Govert J. van Dam, Paul L. A. M. Corstjens, Els Wessels, J. Russell Stothard, Richard Hayes, Helen Ayles, Isaiah Hansingo, Lisette van Lieshout, Amaya L. Bustinduy

**Affiliations:** 1 Department of Clinical Research, London School of Hygiene and Tropical Medicine, London, United Kingdom; 2 MRC Tropical Epidemiology Group, London School of Hygiene and Tropical Medicine, London, United Kingdom; 3 Zambart, Lusaka, Zambia; 4 Department of Parasitology, Leiden University Medical Center, Leiden, The Netherlands; 5 Department of Cell and Chemical Biology, Leiden University Medical Center, Leiden, The Netherlands; 6 Department of Medical Microbiology, Leiden University Medical Center, Leiden, The Netherlands; 7 Department of Tropical Disease Biology, Liverpool School of Tropical Medicine, Liverpool, United Kingdom; 8 Department of Obstetrics and Gynaecology, Livingstone Central Hospital, Livingstone, Zambia; Ghent University, BELGIUM

## Abstract

**Background:**

Given the potentially causal association of female genital schistosomiasis (FGS) with HIV-1 infection, improved diagnostics are urgently needed to scale-up FGS surveillance. The BILHIV (bilharzia and HIV) study assessed the performance of home-based self-collection methods (cervical and vaginal swabs) compared to cervicovaginal lavage (CVL) for the detection of *Schistosoma* DNA by real-time polymerase chain reaction (PCR).

**Methods:**

Between January and August 2018, a consecutive series of female participants from the Population-Cohort of the previous HIV prevention trial HPTN 071 (PopART), resident in Livingstone, Zambia were invited to take part in BILHIV if they were 18–31 years old, non-pregnant and sexually active. Genital self-collected swabs and a urine specimen were obtained and a questionnaire completed at home visits. CVL was obtained at clinic follow-up.

**Results:**

603 women self-collected genital swabs. Of these, 527 women had CVL performed by a mid-wife during clinic follow-up. *Schistosoma* DNA was more frequently detected in genital self-collected specimens (24/603, 4.0%) compared to CVL (14/527, 2.7%). Overall, 5.0% (30/603) women had female genital schistosomiasis, defined as a positive PCR by any genital sampling method (cervical swab PCR, vaginal swab PCR, or CVL PCR) and 95% (573/603) did not have a positive genital PCR. The sensitivity of any positive genital self-collected swab against CVL was 57.1% (95% CI 28.9–82.3%), specificity 97.3% (95.5–98.5%). In a subset of participants with active schistosome infection, determined by detectable urine Circulating Anodic Antigen (CAA) (15.1%, 91/601), positive PCR (4.3%, 26/601), or positive microscopy (5.5%, 33/603), the sensitivity of any positive self-collected specimen against CVL was 88.9% (51.8–99.7%).

**Conclusions:**

Genital self-sampling increased the overall number of PCR-based FGS diagnoses in a field setting, compared with CVL. Home-based sampling may represent a scalable alternative method for FGS community-based diagnosis in endemic resource limited settings.

## Author summary

Female Genital schistosomiasis (FGS) is a neglected and disabling disease that results when eggs from the waterborne parasite *Schistosoma haematobium* are trapped in the human reproductive tract. Current female genital schistosomiasis (FGS) diagnostic strategies are limited because they require expertise and equipment that may not be readily available in low income settings. Improved and accessible diagnostics are urgently needed to scale-up FGS surveillance. This is especially important as FGS has been associated with HIV-1 infection. The BILHIV (bilharzia and HIV) study assessed the performance of home-based self-collection methods (cervical and vaginal swabs) compared with a clinic-based cervicovaginal lavage (CVL) performed by a medical professional. Both methods used real-time polymerase chain reaction (PCR) to detect *Schistosoma* DNA. We found that, in a field setting, self-collected genital and cervical swabs increased the overall number of PCR-based FGS diagnoses, compared with clinically collected CVL. We report the sensitivity of self-collected swabs for the diagnosis of FGS, compared with CVL. We found that the sensitivity of self-collected genital swabs was high in a subset of women with active schistosome infection. We suggest that home-based self-sampling may represent a scalable community-based sampling platform for FGS community-based diagnosis in endemic resource limited settings.

## Introduction

An estimated 82 million women in sub-Saharan Africa live with *Schistosoma (S*.*) haematobium* or *S*. *mansoni* infections [1] that follow fresh water contact. In Zambia alone, it is estimated that 3.8 million people (approximately 22% of the population) are infected with *Schistosoma* species [2]. After maturation, these trematode parasites commence egg-laying in the host’s venous system and disease occurs when tissue-entrapped eggs cause both local and systemic pathology [3]. Female genital schistosomiasis (FGS), defined as the detection of eggs or *Schistosoma spp*. DNA in genital tissue or fluids [4], affects an estimated 20–56 million women worldwide [5], mostly in sub-Saharan Africa, including Zambia [6]. The presence of eggs or *Schistosoma spp*. DNA in urine and stool does not confirm concurrent genital involvement [7, 8].

Egg deposition in FGS occurs in clusters [9], often at the squamocolumnar junction in the subepithelial connective tissue [9] and is therefore frequently missed on superficial Papanicolaou-smear based sampling [8–10]. Often tissue-lodged eggs are accompanied by characteristic sandy patches (both grainy and homogenous) [11] and rubbery papules [12]. However, in up to one-quarter of cases, FGS occurs in macroscopically normal appearing cervical tissue [9, 13]. Classically, FGS lesions are visualized colposcopically [8, 12], requiring equipment and trained clinical expertise that may not be available in low income settings [4]. Also, concern that tissue biopsy, the proposed reference standard, may provide a means of entry for HIV-1 infection [4] has limited its use in FGS research. Given this theoretical risk, the use of polymerase chain reaction (PCR) on cervicovaginal lavage (CVL) has been advocated as an acceptable and less-invasive method of FGS diagnosis in research settings [8, 12, 14, 15]. However, because CVL sampling requires a trained health professional, vaginal speculum insertion, and an intact cold chain, it is not likely scalable for population–based FGS surveillance.

FGS has been associated with adverse reproductive health outcomes, such as infertility [16], ectopic pregnancy [17, 18], and abortion [17]. Eggs deposited in reproductive tissues release immunogenic antigens [19], resulting in granulomatous reaction and fibrosis [18]. There is also increasing evidence on the association of FGS with prevalent HIV-1 infection [10, 20], a relationship that may be causal. Accurate, accessible, and affordable diagnostics are urgently needed to scale-up FGS surveillance and treatment. Schistosome infection is associated with a continuum of disease co-morbidities and post-infection complications [21]. FGS can occur along this continuum and there is currently not a diagnostic test that captures the entire range of FGS clinical presentations [21].

Self-sampling has been used for diagnosis of reproductive tract infections, including human papillomavirus (HPV) [22]. Compared with clinician collected specimens, self-collected PCR-based HPV testing is acceptable to participants [23], increases screening coverage [22, 24], and is as sensitive as clinician collected samples [25]. A home-based diagnostic option for FGS with decreased reliance on medical professionals would have high field applicability. This study was designed to compare the performance of two home-based self-collection methods (cervical and vaginal swabs), to clinic-based, midwife-collected CVL for the detection of *Schistosoma* DNA by Nucleic Acid Amplification Tests (NAATs).

## Methods

### Study site and subjects

Between January and August 2018, participants from the Population Cohort (PC) of HPTN 071 (PopART), a trial to measure the impact of an HIV combination prevention package, including universal HIV test and treat [26], were recruited to participate in the cross-sectional bilharzia and HIV (BILHIV) study. Women were eligible to participate if they were 18–31 years old, non-pregnant, sexually active, and resident in one of the two communities (designated Community-A and Community-B) that participated in HPTN 071 (PopART) in Livingstone, Zambia. Central Livingstone is located within 5–10 kilometres of the Zambezi River, with a tributary flowing in close proximity to Community A. HPTN 071 (PopART) is a cluster randomized trial including 21 participating communities. At the community level, the Population Cohort included one randomly selected adult 18 to 44 years of age from a random sample of households in each community [26]. BILHIV study participants were then selected as a consecutive sample from the HPTN 071 (PopART) Population Cohort. A 2013 survey of school aged children in Livingstone reported prevalence ranges for urinary *S*. *haematobium* infection between 3.3% and 73.3% (median 15.0%, mean 23.3%) [27].

### Home-based sample collection and questionnaire

**Fig 1:**
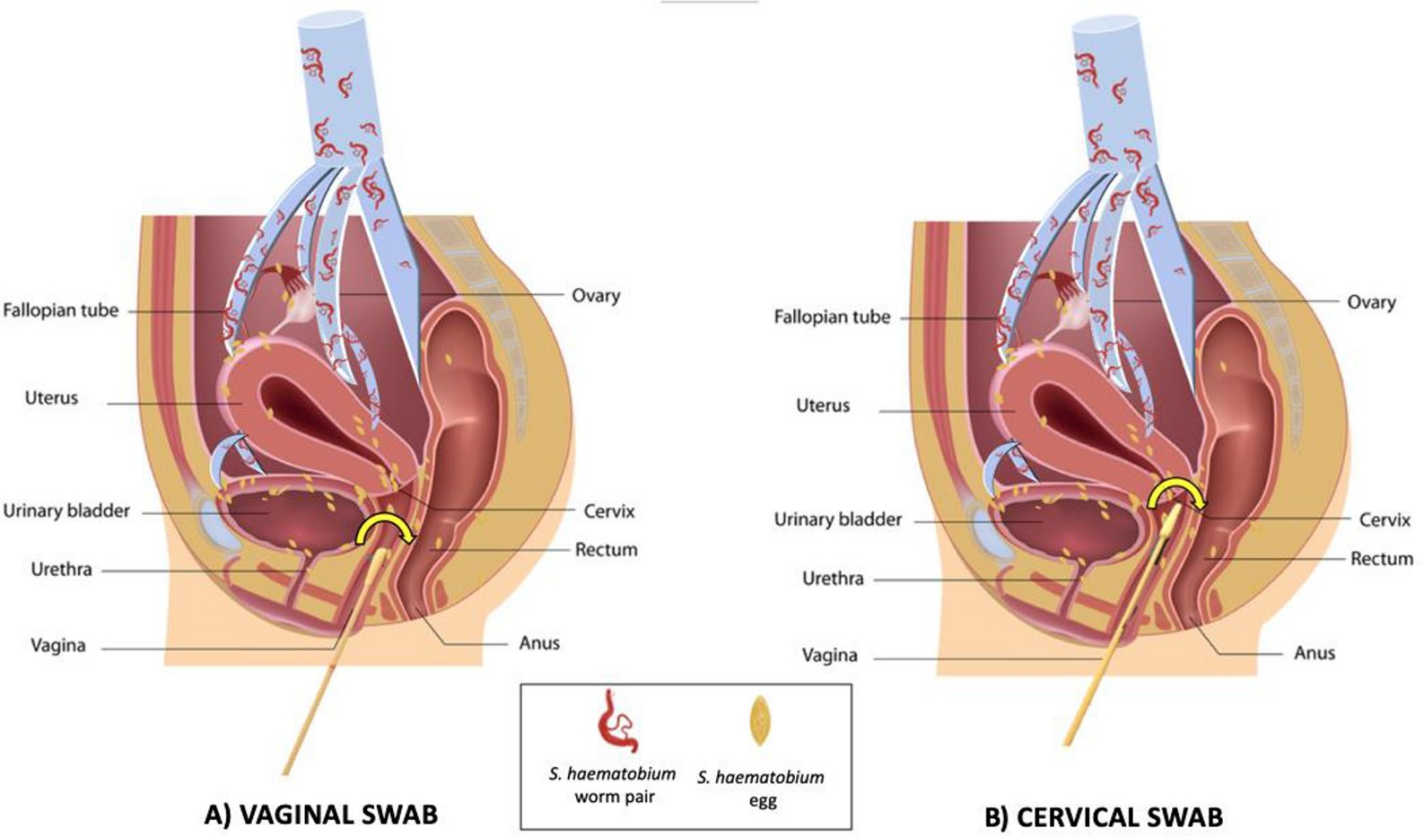
Genital Self-sampling in the BILHIV Study. A) Participants held a 6-inch vaginal swab at the 2 3/8-inch score mark, inserted the swab vaginally until their fingers touched the labia minora, and rotated the swab against the vaginal walls (minimum 15 repetitions). B) Participants held the 6 3/4-inch cervical swab at the non-flocked end, inserted the swab until their fingers touched the labia minora and/or encountered resistance, and rotated the swab against the place of resistance (minimum 15 repetitions).

Trained community workers provided home visits to women who gave an “expression of interest” in the BILHIV study at the HPTN 071 (PopART) PC 36-month visit. The home visit included assessment of eligibility, a questionnaire, genital self-sampling (cervical and vaginal), and a single urine specimen. Trained field workers provided study information in the participant’s preferred language. Following written informed consent, a questionnaire containing questions regarding demographics, water contact, sexual behaviour, and genital symptoms was administered. There were no restrictions on the timing of urine sample self-collection, and 69.5% (419/603) were performed between 9:00 and 14:00. The community worker provided participating women with instructions for urine collection and cervical and vaginal self-sampling. Participants were instructed to hold a 6-inch PrimeSwab (Longhorn Diagnostics, Texas, USA) at the 2 3/8-inch score mark, insert the swab vaginally until their fingers touched the labia minora, and rotate the swab against the vaginal walls (minimum 15 repetitions) (Fig 1). Similarly, for the cervical swab, participants were instructed to hold a 6 3/4-inch flocked swab (Miraclean, Shenzen, China) with a quadrilateral kite-shaped tip at the non-flocked end, insert the swab until their fingers touched the labia minora and/or encountered resistance, and rotate the swab against the place of resistance (minimum 15 repetitions). Each flocked swab head was placed in individual screw cap microtubes (STARLAB, Hamburg, Germany) by the participant after the swab shaft was broken. Both swab specimens and urine were placed in cool boxes for transportation to the laboratory for further processing (see Supplementary Material). Women with evidence of active schistosome infection, defined by any positive urine examination (microscopy, Circulating Anodic Antigen (CAA) or PCR), or women with clinical evidence of FGS as determined by the midwife’s clinical examination [28], were treated free of charge with 40 mg/kg praziquantel, either at the clinic visit, or via community workers.

### Clinic-based sample collection

Enrolled women who were not currently menstruating were invited to attend Livingstone Central Hospital cervical cancer screening clinic within days of self-sampling, where one of two trained midwives performed a cervicovaginal lavage (CVL). After speculum insertion, normal saline (10mL) was flushed continuously with a bulb syringe across the cervix and vaginal walls for one minute and collected from the posterior fornices. Images of the vagina and cervix were captured with a point-of-care colposcope (MobileODT, Tel Aviv, Israel). Participants with suspected reproductive tract infections were offered syndromic management, as per the Ministry of Health [29]. Routine testing for reproductive tract infections was not performed in this study.

### Laboratory analysis

Urine aliquoting for quantification of CAA, PCR, and urinalysis were performed on the day of specimen arrival at the laboratory (see S1 Text). The remaining urine, up to 60mL per participant, was centrifuged in 15mL aliquots and examined by microscopy within 24 hours. The pellet from each 15mL urine aliquot (5 maximum) was evaluated for *S*. *haematobium* eggs. When a pellet contained at least one terminal-spined ovum, the participant was considered positive and the total number of counted eggs in the pellet was reported. Review of all positive and 10% of the negative specimens was conducted blinded by an expert for quality control. Dipsticks were used for analysis of hematuria and proteinuria (Multistix, Siemens, Germany). An up-converting reporter particle (UCP) lateral flow (LF) assay for the quantification of CAA in urine (0.4 mL) was performed at the Leiden University Medical Center (LUMC) (see S1 Text) [30]. Urine CAA antigen levels are known to reflect adult worm burden and decline after successful treatment with praziquantel [31, 32].

### PCR for *Schistosoma spp*

Pre-treatment and DNA isolation of CVL, genital swabs, and urine samples were performed (see S1 Text) in different sites at the LUMC, hence minor differences in the laboratory procedures. Briefly, genital swabs were suspended in PBS and vortexed, and thereafter handled as lavages, by pre-treating using a proteinase K heating step and isolating DNA using QIAamp spin columns (QIAGEN Benelux, Venlo, The Netherlands) [8, 12, 33]. Two hundred μL of urine sample per participant were pretreated using Precellys Soil grinding SK38 (Bertin technology, Montigny-le-Bretonneux, France) and DNA was isolated using MagNA Pure 96 technology (Roche Diagnostics, Penzberg, Germany). Internal transcribed spacer 2-based real-time PCR was performed as previously described [33], with slight modifications (see S1 Text). DNA amplification and detection were performed with the CFX96 Real Time PCR Detection System (BioRad, California, USA). The output in threshold cycles (C_t_) was analysed using BioRad CFX software. Negative and positive control samples were included in each amplification run. Parasite DNA load is categorized by the following pre-specified C_t_ threshold for all specimens processed by PCR (urine, cervical swab, vaginal swab, and CVL): any C_t_-value observed means positive and no C_t_-value observed means negative (S1 Text) [34]. All specimens tested by PCR at LUMC were blinded for clinical and microscopy data.

### Ethical considerations

The study was approved by the University of Zambia Biomedical Research Ethics Committee (reference 011-08-17), the Zambia National Health Research Authority and the London School of Hygiene and Tropical Medicine Ethics Committee (reference 14506). Permission to conduct the study was given by Livingstone District health office and the superintendent of Livingstone Central Hospital. All the human subjects in this study were adults.

### Statistical methods

We anticipated that the prevalence of *S*. *haematobium* infection would be 30% and that PCR performed on self-collected specimens would have sensitivity of 70% and specificity of 85%. Under these assumptions a sample size of 600 women would allow us to detect these target values with 95% confidence intervals of 63%-77% and 81%-88%, respectively.

Data were entered on hand-held electronic data capture devices using Open Data Kit and analysed using STATA 15.1 (Stata Corporation, College Station, TX). Continuous variables were summarized by median and interquartile range (IQR), and categorical variables by frequency and percentage. Differences in participant characteristics between the two communities were evaluated using Fisher’s exact, chi-squared, and Wilcoxon-Mann-Whitney tests. The association between age group and positive test result was assessed using the test for trend. For the comparison of home-based self-collection methods with clinic based CVL, we calculated sensitivity and specificity. There weren’t indeterminate PCR results or missing data for genital self-collected swabs (the index test). “Definite” FGS was defined as any positive genital PCR (cervical swab, vaginal swab or CVL). The presence of either CAA or *S*. *haematobium* eggs in clinical specimens indicates the presence of infection with viable worms [30]. Thus, in this study, “active” schistosome infection refers to participants with a positive urine PCR, CAA, or microscopy. To evaluate if sequential use of FGS diagnostics might be beneficial, an ad-hoc secondary analysis was performed in which calculation of the diagnostic performance was restricted to those with an active schistosome infection according to positive urine PCR, CAA, or microscopy. CVL was chosen as the *a priori* reference standard for the BILHIV study. In the absence of a universally recognized reference standard for FGS, a composite FGS outcome was constructed, defined as a positive result on any genital PCR specimen (swab or CVL).

## Results

**Fig 2:**
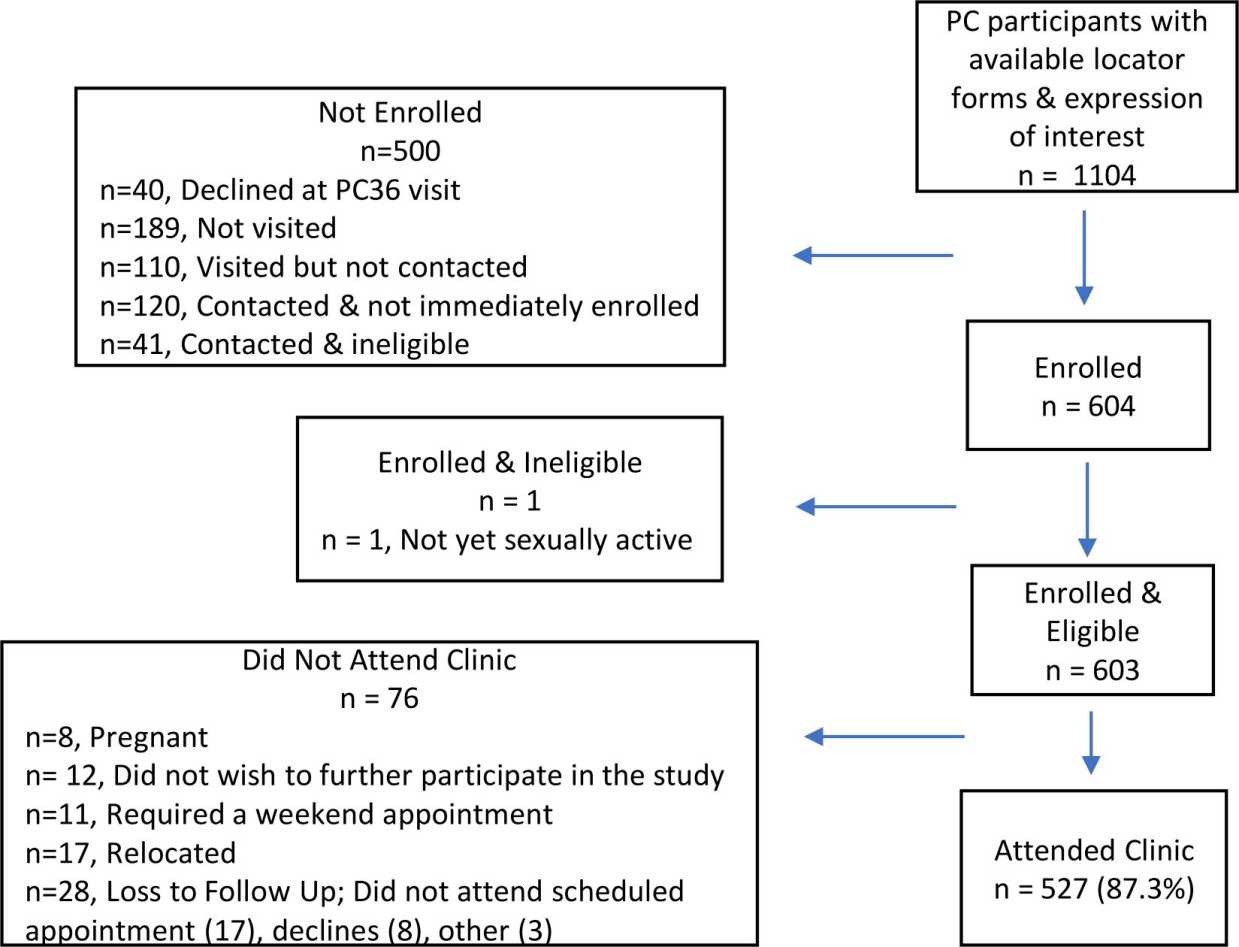
BILHIV study flow diagram. Not visited - the participant was not visited before the study closed for enrollment (189) Visited but not contacted - a visit was made to the study household, but the participant could not be located (70), had relocated (39), or died (1) Contacted & not immediately enrolled - (42), out of town (18), declined to participate (60) Contacted & ineligible - virgin (16), pregnant (17), over 31 (8)

Overall, 1105 women in the study communities from the HPTN 071 (PopART) Population Cohort met the inclusion criteria. A total of 603 (54.5%) eligible women were enrolled with 527 (87.0%) completing clinic follow up (Fig 2). Women from both communities reported regular water contact in childhood (p = 0.22) (Table 1). Overall, an active schistosome infection was diagnosed in 15.6% (94/601) (Table 2), of which 33 were urine microscopy positive and an additional 61 cases were diagnosed by urine CAA. Urine PCR confirmed the presence of schistosome infection in 26 participants and did not detect any additional cases. Overall, 15.6% (94/601) had schistosome infection (Table 2). The prevalence of *S*. *haematobium* infection was 5.5% (33/603) based on urine microscopy, 15.1% (91/601) by urine CAA, and 4.3% (26/601) by urine PCR (Table 2).

**Table 1:** Baseline characteristics of 603 Zambian women living in *S*. *haematobium* endemic areas near the Zambezi river by district.

Characteristics		Overall (n = 603)	Community A (n = 319)	Community B (n = 284)	p-value*
Age in years–Median (IQR)		24 (22–28)	26 (23–29)	24 (21–27)	<0.001
Marital Status	Single	258 (42.8%)	110 (34.5%)	148 (52.1%)	<0.001
	Married or Cohabitating	320 (53.1%)	193 (60.5%)	127 (44.7%)	
	Divorced or Separated	23 (3.8%)	15 (4.7%)	8 (2.8%)	
	Widowed	2 (0.3%)	1 (0.3%)	1 (0.4%)	
Education (highest level)	Any Primary School	167 (27.7%)	117 (36.7%)	50 (17.6%)	<0.001
	Any Secondary School	364 (60.4%)	173 (54.2%)	191 (67.3%)	
	Training in a Trade	59 (9.8%)	20 (6.3%)	39 (13.7%)	
	Degree or Higher	3 (0.5%)	3 (0.9%)	0 (0.0%)	
	None	10 (1.7%)	6 (1.9%)	4 (1.4%)	
Employment Status	Working	408 (67.7%)	200 (62.7%)	208 (73.2%)	0.006
	Not Working	195 (32.3%)	119 (37.3%)	76 (26.8%)	
Current Water Contact	None	512 (84.9%)	263 (82.5%)	249 (87.7%)	0.02
	At Least Weekly	18 (3.0%)	11 (3.5%)	7 (2.5%)	
	Every 1–2 Months	30 (5.0%)	24 (7.5%)	6 (2.1%)	
	Every 6–12 Months	43 (7.1%)	21 (6.6%)	22 (7.8%)	
Childhood Water Contact	None	186 (30.9%)	96 (30.1%)	90 (31.7%)	0.22
	At Least Weekly	381 (63.2%)	208 (65.2%)	173 (60.9%)	
	Every 1–2 Months	24 (4.0%)	12 (3.8%)	12 (4.2%)	
	Every 6–12 Months	12 (2.0%)	3 (0.9%)	9 (3.2%)	
Self-reported History of Schistosomiasis	No	572 (94.8%)	294 (92.2%)	278 (97.9%)	0.006
	Yes	25 (4.2%)	20 (6.3%)	5 (1.8%)	
	Maybe	6 (1.0%)	5 (1.6%)	1 (0.4%)	
Self- reported Treatment with Praziquantel	No	523 (86.7%)	270 (84.6%)	253 (89.1%)	0.11
	Yes	61 (10.1%)	40 (12.5%)	21 (7.4%)	
	Maybe	19 (3.2%)	9 (2.8%)	10 (3.5%)	

* comparison of Community-A vs Community-B

**Table 2:** Diagnostic test results and FGS prevalence in 603 Zambian women living in *S*. *haematobium* endemic areas near the Zambezi river.

Characteristics	Overall (n = 603) % (n)	Community-A (n = 319) % (n)	Community-B (n = 284) % (n)	p-value
Hematuria	28.7 (173)	31.0 (99)	26.1 (74)	0.2
Urine Microscopy	5.5 (33/603)	8.2 (26/319)	2.5 (7/284)	0.002
*Median egg count per 15mL*^*‡*^	18	17.5	18	
*IQR*	5–35	5–35	8–90	
Urine CAA*	15.1 (91/601)	20.5 (65/317)	9.2 (26/284)	<0.001
*Median pg/mL*^*‡*^	9.10	11.46	5.93	
*IQR*	2.2–58.6	2.3–42.1	2.2–112.0	
Urine PCR*	4.3 (26/601)	6.3 (20/317)	2.1 (6/284)	0.012
*Median Ct*^*‡*^	29.6	30.2	29.3	
*IQR*	27.8–33.5	26.4–34.9	29.0–32.7	
Active schistosome Infection* [any urine test positive]	15.6 (94/601)	21.1 (67/317)	9.5 (27/284)	<0.001
PCR–CVL^†^	2.7 (14/527)	3.8 (11/290)	1.3 (3/237)	0.06**
*Median Ct*^*‡*^	34.6	35.3	33.2	
*IQR*	33.0–37.0	33.0–37.0	21.0–38.0	
PCR–Cervical Swab	3.3 (20/603)	4.4 (14/319)	2.1 (6/284)	0.12
*Median Ct*^*‡*^	35.3	35.8	33.3	
*IQR*	29.6–37.1	29.4–37.2	29.7–36.3	
PCR–Vaginal Swab	2.5 (15/603)	2.5 (8/319)	2.5 (7/284)	0.97
*Median Ct*^*‡*^	34.3	35.4	32.2	
*IQR*	23.6–37.1	30.6–37.2	23.2–35.2	
Any PCR Positive Sample^††^	5.7 (30/529)	7.6 (22/291)	3.4 (8/238)	0.02
PCR positive–Vaginal or Cervical Swab	4.0 (24/603)	5.3 (17/319)	2.5 (7/284)	0.07

*2 urine vials arrived at LUMC empty (n = 601)

** Calculated by Fisher’s exact (otherwise by chi-squared)

^†^ 527 women presented for CVL– 290 Community-A and 237 Community-B

^*‡*^ Median egg count, CAA concentrations and PCR Ct-value restricted to positive tests

^††^One participant from each of Site-1 and Site-2 had positive self-collected PCR specimens but did not present to clinic (n = 529)

**Abbreviations:** CAA—Circulating Anodic Antigen, CVL–Cervicovaginal Lavage, PCR–Polymerase Chain Reaction for the detection of *Schistosoma* DNA

**Fig 3:**
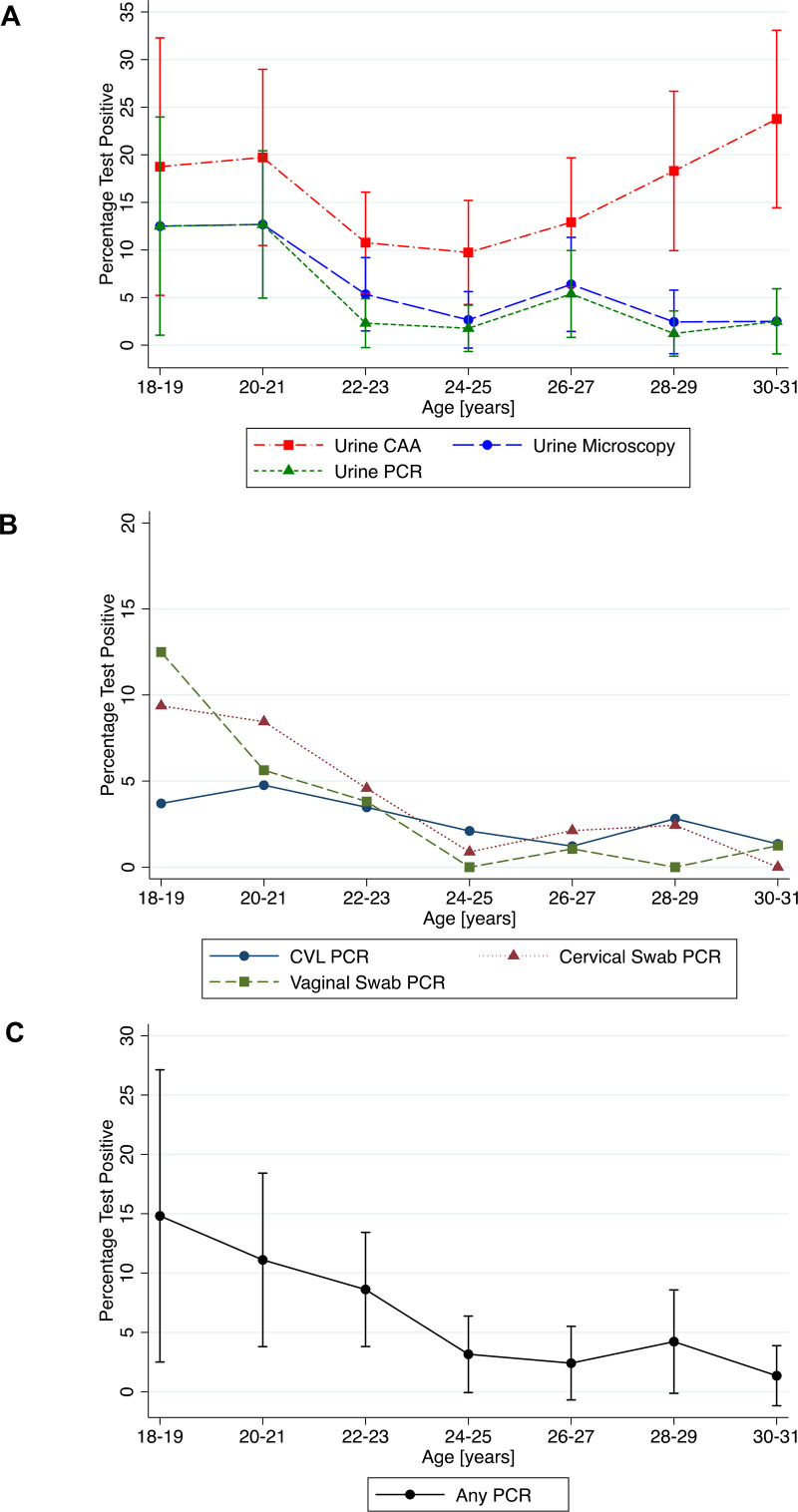
Distribution of positive diagnostic test results by age group. A. Distribution of positive urine CAA, urine microscopy, and urine PCR results by age group^*^ B. Distribution of positive genital PCR results by age group^*^ C. Distribution of any positive genital PCR result by age group* *Please see S1 Table for further details of the numbers of women tested per time point

**Fig 4:**
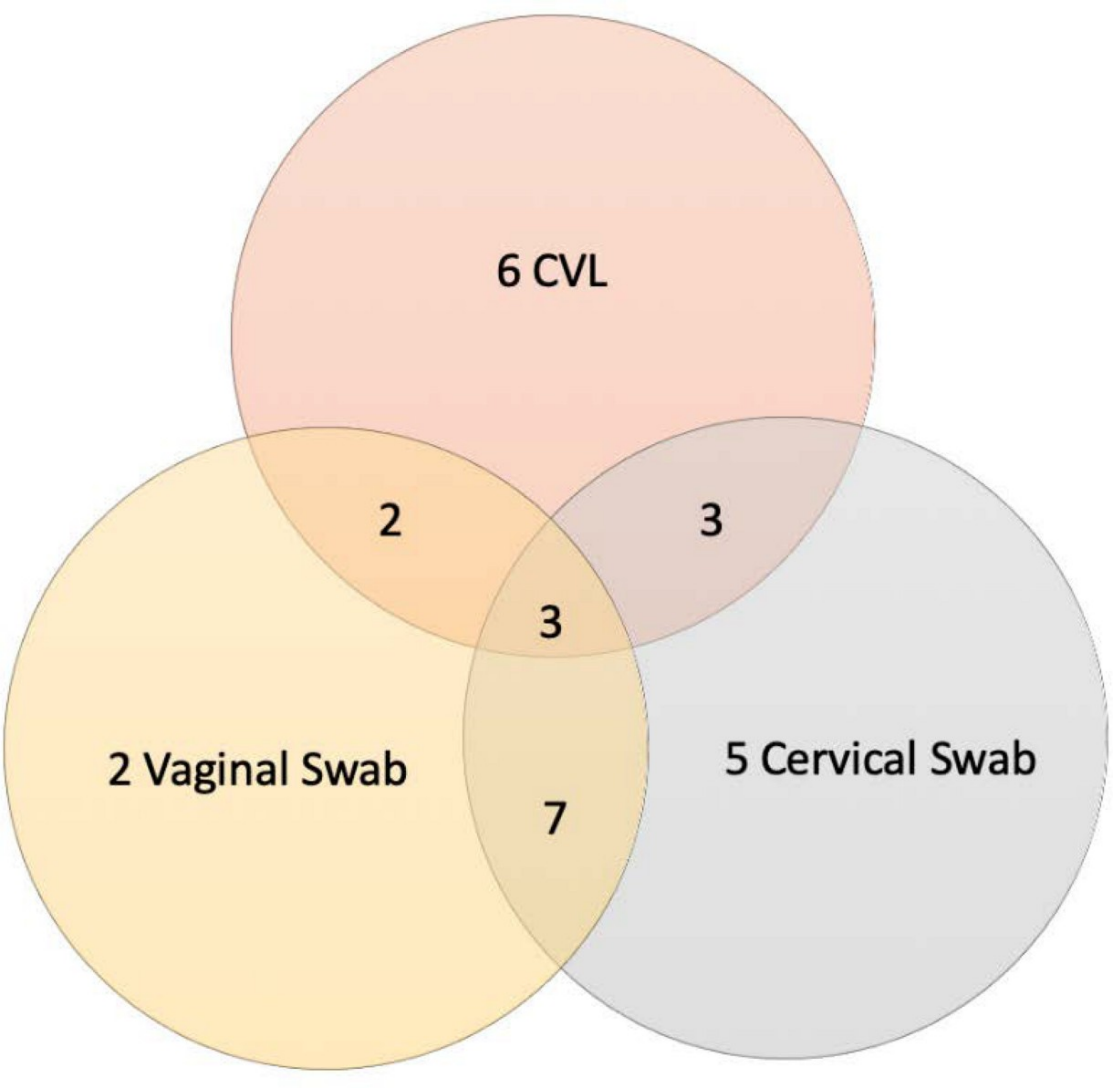
Venn Diagram of 28* Positive PCR-based Self-Collected Specimens (paired with CVL) for *Schistosoma* by Collection Type *2 participants with positive genital self-collected specimens did not follow up in clinic for cervicovaginal lavage.

There were 30 women (5.0%) with definite FGS, defined as any positive genital PCR (cervical swab, vaginal swab or CVL), 22/291 (7.6%) in Community-A and 8/238 (3.4%) in Community-B (p = 0.04) and 95% (573/603) did not have definite FGS. The proportion of participants testing positive decreased with increasing age for all tests except urine CAA and CVL PCR (urine microscopy p-trend = 0.004, urine PCR p-trend = 0.003, any PCR-positive genital specimen p-trend<0.001, cervical swab PCR p-trend<0.001, vaginal swab PCR p-trend<0.001) (Fig 3 & S1 Table). In the 30 women with FGS, the prevalence of active schistosome infection was 70.0% (21/30). Of the 94 women with an active schistosome infection, 22.3% (21/94) had FGS and of the 507 women without active schistosome infection 1.8% (9/507) had FGS (p<0.001), these numbers include women who did not attend clinic follow-up. Fig 4 illustrates FGS diagnosis based on PCR positivity for each of the three genital sampling methods. No adverse events were reported in either group related to use of the index or reference test.

The sensitivity of a positive result from any positive self-collected specimen (cervical or vaginal swab) against CVL was 57.1% (28.9–82.3) with specificity of 97.3% (95.5–98.5%) (Table 3). Compared with CVL, the sensitivity of self-collected cervical swabs (42.9% [17.7–71.1%]) was somewhat higher than for vaginal swabs (35.7% [12.8–64.9]), albeit with wide confidence intervals. The specificity of PCR in self-collected specimens versus CVL was high regardless of sampling technique (cervical 97.7% [95.9–98.8%], vaginal 98.2% [96.7–99.2%]) (Table 3). Self-collected genital swabs were also evaluated as the reference standard (Table 3). Of these comparisons, the highest sensitivity (80% [61.4–92.3] was achieved using combined swab results compared with the composite FGS diagnosis (Table 3).

**Table 3:** Sensitivity and specificity of genital specimens for the detection of *Schistosoma* DNA by PCR.

Reference Standard	Index test	Sensitivity (%)	Specificity (%)
Cervicovaginal lavage PCR	Vaginal swab PCR	35.7 [5/14] (12.8–64.9)	98.2 [504/513] (96.7–99.2)
	Cervical swab PCR	42.9 [6/14] (17.7–71.1)	97.7 [501/513] (95.9–98.8)
	Vaginal or Cervical Swab PCR	57.1 [8/14] (28.9–82.3)	97.3 [499/513] (95.5–98.5)
Cervical swab PCR	Cervicovaginal Lavage PCR	33.3 [6/18] (13.3–59.0)	98.4 [501/509] (96.9–99.3)
	Vaginal Swab PCR	55.0 [11/20] (31.5–76.9)	99.3 [579/583] (98.3–99.8)
Vaginal Swab PCR	Cervicovaginal Lavage PCR	35.7 [5/14] (12.8–64.9)	98.2 [504/513] (96.7–99.2)
	Cervical Swab PCR	73.3 [11/15] (44.9–92.2)	98.5 [579/588] (97.1–99.3)
Vaginal or Cervical Swab PCR	Cervicovaginal Lavage PCR	36.4 [8/22] (17.2–59.3)	98.8 [499/505] (97.4–99.6)
Composite FGS Diagnosis*	Vaginal swab PCR	50.0 [15/30] (31.3–68.7)	100.0 [499/499] (99.3–100.0)
	Cervical swab PCR	66.7 [20/30] (47.2–82.7)	100.0 [499/499] (99.3–100.0)
	Vaginal or Cervical Swab PCR	80.0 [24/30] (61.4–92.3)	100.0 [499/499] (99.3–100.0)
	Cervicovaginal Lavage PCR	50.0 [14/28] (30.6–69.4)	100.0 [499/499] (99.3–100.0)

**Abbreviations:** CAA–Circulating Anodic Antigen, CS–Cervical Swab, CVL–Cervicovaginal Lavage, PCR–Polymerase Chain Reaction for the detection of *Schistosoma* DNA, VS–Vaginal Swab

*Composite FGS Diagnosis–any positive PCR result on a genital specimen (CVL, vaginal swab or cervical swab)

In a secondary analysis of those participants diagnosed with an active schistosome infection, the sensitivity of PCR from any positive self-collected swab specimen (cervical or vaginal) against CVL was marginally higher than in the primary analysis, albeit with wide confidence intervals (Table 4). The gain in sensitivity when evaluating those participants diagnosed with an active schistosome infection was accompanied by a decline in specificity (Table 4).

**Table 4:** Sub-analysis of sensitivity and specificity of self-collected genital swabs compared with cervicovaginal lavage for the detection of *Schistosoma* DNA in participants with positive urine specimens: CAA(n = 78), microscopy (n = 32), PCR (n = 26).

Reference test	Index test	Sensitivity (%)	Specificity (%)
**Urine CAA**			
Cervicovaginal lavage PCR	Vaginal swab PCR	50.0 [4/8] (15.7–84.3)	90.0 [63/70] (80.5–95.9)
	Cervical swab PCR	62.5 [5/8] (24.5–91.5)	87.1 [61/70] (77.0–93.9)
	Vaginal or cervical swab PCR	87.5 [7/8] (47.3–99.7)	85.7 [60/70] (75.3–92.9)
**Urine Microscopy**			
Cervicovaginal lavage PCR	Vaginal swab PCR	55.6 [5/9] (21.2–86.3)	73.9 [17/23] (51.6–89.3)
	Cervical swab PCR	66.7 [6/9] (29.9–92.5)	56.5 [13/23] (34.5–76.8)
	Vaginal or cervical swab PCR	88.9 [8/9] (51.8–99.7)	56.5 [13/23] (34.5–76.8)
**Urine PCR**			
Cervicovaginal lavage PCR	Vaginal swab PCR	55.6 [5/9] (21.2–86.3)	70.6 [12/17] (44.0–89.7)
	Cervical swab PCR	66.7 [6/9] (29.9–92.5)	47.1 [8/17] (23.0–72.2)
	Vaginal or cervical swab PCR	88.9 [8/9] (51.8–99.7)	47.1 ([8/17] 23.0–72.2)
**Active Schistosome Infection (Urine positive for PCR, CAA, or Microscopy)**			
Cervicovaginal lavage PCR	Vaginal swab PCR	55.6 [5/9] (21.2–86.3)	90.3 [65/72] (81.0–96.0)
	Cervical swab PCR	66.7 [6/9] (29.9–92.5)	86.1 [62/72] (75.9–93.1)
	Vaginal or cervical swab PCR	88.9 [8/9] (51.8–99.7)	84.7 [61/72] (74.3–92.1)

## Discussion

The BILHIV study is the first to examine the performance of self-collected genital swabs (vaginal and cervical) for the diagnosis of FGS. Self-collected swabs were compared with provider-collected CVL, an accepted, non-invasive standard for FGS diagnosis in research settings [12, 14]. The addition of self-collected swabs to CVL increased the number of FGS diagnoses. The sensitivity of any self-collected genital specimen compared with CVL improved when only those diagnosed with an active infection (i.e. positive urine CAA, urine PCR, or urine microscopy) were considered. Using a composite definition of FGS (any PCR positive genital specimen) as the reference standard also improved the sensitivity of self-collected genital specimens. In the absence of a reference standard for FGS [21], the specificity of genital swabs is limited by comparison with CVL alone. CVL for FGS diagnosis has imperfect sensitivity and may not identify all true positives. In this analysis, the sensitivity of CVL as the index test compared to self-sampling as the reference was similar to that of self-sampling as the index test with CVL as reference standard.

A sub-analysis of the BILHIV data also suggests that sensitivity of PCR-based testing of genital self-samples is high in the subgroup of participants with active infection. The cost of genital swabs (0.50$/vaginal swab and 0.30$/cervical swab) and molecular testing (6.68$/test) may be affordable in some research settings, but more field-appropriate and scalable NAAT methods should be investigated. Given the cost of FGS diagnostics, an algorithm which advocates FGS screening in resource-limited settings among women with positive CAA or urine microscopy could assist in conserving resources, labour, reagents, and other costs. Applying this algorithm to the BILHIV study identifies 70.0% of FGS cases identified in a relatively low-prevalence area. However, an important limitation of restricting genital self-sampling to a sub-group with active infection is a decreased ability to detect FGS cases in the population, as FGS would not be identified in women without active infection. This decrement in the overall sensitivity of screening must be balanced against the availability of resources. Other practical limitations to this strategy include low prevalence populations, the imperfect sensitivity of *Schistosoma* PCR in genital self-sampling, and the current availability of PCR and CAA as research tools that do not yet allow individual rapid diagnosis at the point-of-care [35]. To maximally leverage resources, FGS self-sampling would be integrated into other reproductive health platforms, ideally those that also utilize self-sampling in the diagnosis of reproductive tract infections, such as HPV [22, 25].

Scalable, affordable, and field-acceptable diagnostics are necessary to improve our understanding of the current population prevalence of FGS and to limit the negative health impact of HIV-1, infertility, and gynaecologic symptoms. After repeated fresh-water exposures, girls living in endemic areas may develop the genital changes associated with FGS prior to sexual debut or the onset of menstruation [36]. A single dose treatment of praziquantel will decrease egg production. but will only kill a proportion of living schistosomes [3]. Data on the effect of praziquantel treatment in FGS are limited, with small numbers and heterogeneous methods, but do suggest a proportion of FGS lesions remain despite praziquantel treatment [37–39]. Thus, early identification of FGS and subsequent praziquantel treatment prior to the age of 21 is associated with beneficial outcomes, such as lower prevalence of contact bleeding, lower odds of sandy patches [40], and lower rates of sub-fertility [41]. Prevention efforts cannot be underestimated, and young women should be prioritized to obtain the maximum benefit from the roll-out of FGS diagnosis and treatment.

To date, no previous studies of FGS diagnostics have used CVL as a reference standard and cross-study comparisons are limited due to heterogeneity of methods [8, 12, 14, 21]. Previously published work has reported low sensitivity and high specificity of *Schistosoma* CVL PCR for FGS diagnosis in African populations [8, 14, 21]. In adult Zimbabwean women (ages 15–49), CVL PCR sensitivity was 53% and specificity 79% compared with cervical visualization and histopathology [14]. The study had some limitations relating to loss of specimens due to cold chain malfunctions, and extended specimen storage time (10 years), potentially affecting PCR outcomes. In an adolescent South African population (age range 15–23), CVL PCR was compared with urine microscopy, cervical visualization, and urogenital symptoms by latent class analysis with a sensitivity of 52.4% (33.2–73.6) and specificity of 42.4% (37.9–47.0) [21]. Together, these studies further illustrate the limited performance of *Schistosoma* PCR in the absence of a reliable FGS reference standard [15].

The performance of diagnostic tests for schistosome infection can vary by background parasite prevalence [42]. Important information regarding disease stage, intensity and morbidity may be missed in the absence of appropriate diagnostics [31]. No one test captures all characteristics of infection simultaneously and with perfect sensitivity. Conventional diagnostic tests (reagent strips and urine microscopy) are insensitive, especially in low-prevalence areas, where light intensity infections can be underestimated [42]. The overall prevalence of schistosome infection in our study population was 5.5% by single urine microscopy, meeting the WHO classification of a low-risk community [43]. Egg detection in urine microscopy may increase slightly with serial urine collections [7, 44]. The prevalence of FGS, as diagnosed by a positive PCR in any genital specimen, was 5.0% with site variations (2.8%-6.9%). Our study took place in two communities within Livingstone. Community-A is closer to a water source and women in Community-A had a higher overall prevalence of schistosome infection and FGS. The sensitivity of microscopy, urine PCR, and CAA diminish in areas of low parasite prevalence [42], or after treatment with praziquantel [44]. Sensitivity of CAA can be increased by using a larger sample volume [45]. Our results confirm previous findings that urine CAA has higher sensitivity than microscopy in low-endemic settings [42] and further illustrates that schistosome infection is focal and prevalence can vary within neighbouring communities.

When comparing the performance of sampling techniques for parasite DNA retrieval, cervical swabs detected 5 cases of FGS not detected by vaginal swabs and CVL. This was also true for vaginal swabs (2 FGS cases) and CVL (6 FGS cases) that were not detected in other sites examined by PCR-based methods (Fig 4). In the absence of self-collecting cervical cytology, it is not possible to confirm if the participants self-sampled from the cervix. However, the longer swab and collection techniques likely allowed a high cervicovaginal specimen that contributed to a greater number of PCR positive samples than vaginal swabs. Studies of FGS histopathology report the most common location for egg deposition is the cervix [6], which may explain the higher proportion of positive specimens in specimens detecting DNA from this site. Genital swabs are also field-appropriate, acceptable to participants, and scalable, while CVL requires vaginal speculum insertion and is performed by trained health workers.

The BILHIV study was conducted in a low prevalence area, therefore the presented estimates of sensitivity are subject to a high degree of imprecision due to the small numbers of total FGS cases. There was excellent retention with 87.4% (527/603) of participants presenting to the clinic for CVL, with very little missing data. The women self-collected vaginal and cervical specimens privately in their own homes. This raises the question of false negative genital swabs, which could be addressed by measuring B-globin PCR as a positive control to confirm the presence of human DNA [22]. BILHIV data confirm previous findings that *Schistosoma* DNA can be detected in the genital tract in the absence of egg excretion [7, 8], and in participants with negative CAA. While these specimens may appear as false positives, the reported specificity of PCR for detecting *Schistosoma* DNA is near 100% [14, 21]. *S*. *haematobium* eggs can be detected in semen [46] and our study did not objectively evaluate for PSA or other markers of recent sexual contact.

Overall, the relatively high concordance of DNA detection in genital self-collected specimens and CVL suggest that self-collection methods for the diagnosis of FGS are feasible in resource limited areas. Drawing on the experience of HPV, self-sampling has been shown to be scalable and effective in real-world settings [47]. As the focus in schistosomiasis shifts from morbidity control to elimination, data on the performance of diagnostic tests for infection and morbidity in low-prevalence settings becomes increasingly applicable [48]. To achieve elimination of both infection and chronic disease such as FGS, low-prevalence areas require novel and innovative interventions and diagnostic strategies to provide site-appropriate, accurate prevalence estimates [48].

## Conclusion

Genital self-sampling increased the overall number of PCR-based FGS diagnoses in a field setting, compared with cervicovaginal lavage. Schistosomiasis is focal and background parasite prevalence may impact test sensitivity. Home-based self-sampling may represent a scalable alternative method for FGS community-based diagnosis in endemic resource limited setting.

## Supporting information

S1 Text Supplementary materials and methods. (DOCX)

S1 Table Positive *Schistosoma* diagnostic test results by age. (DOCX)
