# Supplementary material for: Genital self-sampling compared with cervicovaginal lavage for the diagnosis of female genital schistosomiasis in Zambian women: The BILHIV study
Source: PLoS Negl Trop Dis. 2020 Jul 14;14(7):e0008337. doi: 10.1371/journal.pntd.0008337 (PMC7360036; doi:10.1371/journal.pntd.0008337)
Supplement: S1 Text — (DOCX) [file pntd.0008337.s001.docx]

**S1 Text - Supplementary Materials and Methods**

**Storage and transport of samples**

The urine aliquots and swab specimens were immediately stored at -80°C after arrival at the laboratory, while the CVL fluid was placed immediately on ice after collections in the clinic and thereafter also stored at -80°. All samples were transported under frozen conditions to the Netherlands for further testing at the Leiden University Medical Center (LUMC). All laboratory analysis was performed blinded from any other data.

**Circulating Anodic Antigen**

Circulating Anodic Antigen is excreted from the gut of live schistosome worms into the host’s bloodstream during active infection. An up-converting phosphor (UCP) lateral flow (LF) assay for CAA was performed on the collected urine specimens at the LUMC department of Cell and Chemical Biology, as previous described [1, 2]. CAA comprises repetitive carbohydrate epitopes that efficiently bind the UCP reporter and the test-line on the LF strip via a CAA specific mouse monoclonal antibody. Interfering proteins were removed with a trichloroacetic acid (TCA) extraction by mixing 400 μL urine with 100 μL 12% (wt/vol) TCA. A centrifugation step removed the precipitate and 500 μL of the resulting clear TCA supernatant was concentrated to 20 μL using an 0.5 mL Amicon filtration device (Merck Inc.). Subsequently, the concentrate was mixed with a high salt lateral flow buffer containing the UCP reporter conjugated with monoclonal mouse anti-CAA antibody and incubated at 37°C for one hour. The same monoclonal mouse anti-CAA antibody is immobilized on a test line on a nitrocellulose membrane of an LF strip. These LF strips, comprised of a glass fiber sample pad, a nitrocellulose membrane, and an absorbent pad are inserted into microtiter plate wells with specimen for immunochromatography [1]. UCP technology uses a luminescent reporter particle that emits light upon excitation with 980 nm infrared light. Finally, LF strips are read with a modified Packard Fluorocount microtiter plate reader suited for IR excitation and LF strips. Measured signals are compared to a standard series with known amounts of CAA. For this assay, analyzing the equivalent of 417 μL urine, a CAA value of 0.6 pg/mL was considered positive based on a series of negative controls (highest value plus 2 SDs).

**Detection of *Schistosoma* DNA**

The internal transcribed spacer 2–based real-time PCR was performed for the detection of *Schistosoma* DNA in the clinical samples [3], with some minor modifications described below. This PCR, using schistosome primers Ssp48F and Ssp124R and the double labeled probe Ssp78T, has been extensively validated on its specificity [4-6]. Appropriate positive and negative controls were included at each PCR run and, in addition, an internal control (Phocin herpes virus 1 (PhHV-1),10^3^ PFU/mL)) was added to each sample reaction for detection of potential inhibition of amplification [3]. For all specimens, DNA amplification and detection were performed with the CFX96 Real Time PCR Detection System (BioRad, California, USA). The output in threshold cycles (Ct) was analysed using BioRad CFX software.

**Urine DNA isolation and PCR**

Urine PCR was performed at the department of Medical Microbiology of the LUMC. A 200 µl aliquot of each urine sample was transferred to a 2 ml tube containing Precellys Soil grinding SK38 (Bertin technology Montigny-le-Bretonneux, France) and 1.25 ml STAR buffer (Roche). The tubes were mixed in the Precellys 24 tissue homogeniser (Bertin instruments, Montigny-le-Bretonneux, France) on 5500 rpm for 10 seconds, followed by a 1minute incubation. Centrifugation at 5500rpm was repeated, followed by a two minute incubation in the machine. The tubes were incubated 5 minutes at room temperature and centrifuged for 1 minute at 14000 rpm in an Eppendorf centrifuge. A 200 µl sample was transferred to a MagNA Pure 96 cartridge and nucleic acids were isolated on the MagNA Pure 96 instrument (Roche Diagnostics, Penzberg, Germany) using the MagNA Pure 96 DNA and Viral NA small volume kit with PhHV as internal control. The PCR conditions were slightly adapted from those described previously [3]. In the BILHIV study, 10 µl DNA sample was used for the PCR and the PCR conditions were 15 minutes 95^o^C, followed by 45 cycles of 5 seconds 95^o^C, 15 seconds 55^o^C and 15 seconds 72^o^C.

**Genital DNA isolation and PCR**

Genital PCR was performed at the department of Parasitology of the LUMC. After thawing, 1.5 mL of PBS was added to each tube containing a swab. After being vortexed for 10 seconds, the swabs were left at room temperature for another hour, after which the fluids were processed similar to the cervicovaginal lavage fluid. DNA extraction and PCR set up was performed at LUMC using a custom automated liquid handling station (Hamilton, Switzerland) [4]. DNA was extracted from 200uL of specimen using a proteinase K heating step followed by QIAamp spin columns (QIAGEN Benelux; Venlo, The Netherlands). Amplification consists of 15 min at 95°C followed by 50 cycles of 15 seconds at 95°C, 30 seconds at 60°C, and 30 seconds at 72°C.

**Cross tabulation of cervical and vaginal swab results (index test) by results of the cervicovaginal lavage (reference standard)**

|  | Cervicovaginal lavage positive | Cervicovaginal lavage negative |  |
| --- | --- | --- | --- |
| Any genital swab positive | 8 | 14 | 22 |
| Any genital swab negative | 6 | 499 | 505 |
|  | 14 | 513 | 527 |

**References:**

1. Corstjens PL, de Dood CJ, Kornelis D, et al. Tools for diagnosis, monitoring and screening of *Schistosoma* infections utilizing lateral-flow based assays and upconverting phosphor labels. *Parasitology* 2014; **141**(14): 1841-55.
2. Corstjens PL, de Dood CJ, Knopp S, et al. Circulating Anodic Antigen (CAA): A highly sensitive diagnostic biomarker to detect active *Schistosoma* infections – improvement and use during SCORE American Journal of Tropical Medicine and Hygiene (in press) **2019**
3. Obeng BB, Aryeetey YA, de Dood CJ, et al. Application of a circulating-cathodic-antigen (CCA) strip test and real-time PCR, in comparison with microscopy, for the detection of *Schistosoma* haematobium in urine samples from Ghana. Annals of tropical medicine and parasitology **2008**; 102:625-33
4. Pillay P, van Lieshout L, Taylor M, et al. Cervical cytology as a diagnostic tool for female genital schistosomiasis: Correlation to cervical atypia and *Schistosoma* polymerase chain reaction. CytoJournal **2016**; 13:10.
5. Randrianasolo BS, Jourdan PM, Ravoniarimbinina P, et al. Gynecological manifestations, histopathological findings, and *schistosoma*-specific polymerase chain reaction results among women with *Schistosoma* *haematobium* infection: a cross-sectional study in Madagascar. The Journal of infectious diseases **2015**; 212:275-84.
6. Vinkeles Melchers NV, van Dam GJ, Shaproski D, et al. Diagnostic performance of Schistosoma real-time PCR in urine samples from Kenyan children infected with *Schistosoma haematobium*: day-to-day variation and follow-up after praziquantel treatment. PLoS Negl Trop Dis **2014**; 8:e2807
