# Supplementary material for: Genital self-sampling compared with cervicovaginal lavage for the diagnosis of female genital schistosomiasis in Zambian women: The BILHIV study
Source: PLoS Negl Trop Dis. 2020 Jul 14;14(7):e0008337. doi: 10.1371/journal.pntd.0008337 (PMC7360036; doi:10.1371/journal.pntd.0008337)
Supplement: S1 Table — (DOCX) [file pntd.0008337.s002.docx]

**S1 Table – Positive *Schistosoma* Diagnostic Test Results by Age**

| **Age** | **N (%)** | **Urine**  **CAA**  **% (n)** | **Urine**  **Microscopy**  **% (n)** | | **Urine**  **PCR**  **% (n)** | **Active Infection***^**^*  **% (n)** | **Cervical Swab PCR % (n)** | **Vaginal**  **Swab PCR**  **% (n)** | **Vaginal Lavage PCR**  **% (n)** | **Any Positive Genital PCR**  **% (n)** |
| --- | --- | --- | --- | --- | --- | --- | --- | --- | --- | --- |
| **18-19** | **32 (5.3)** | **18.8 (6/32)** | | **12.5 (4/32)** | **12.5 (4/32)** | **18.8 (6/32)** | **9.4 (3/32)** | **12.5 (4/32)** | **3.7 (1/27)** | **14.8 (4/27)** |
| **20-21** | **71 (11.8)** | **19.7 (14/71)** | | **12.7 (9/71)** | **12.7 (9/71)** | **19.7 (14/71)** | **8.5 (6/71)** | **5.6 (4/71)** | **4.8 (3/63)** | **11.1 (7/63)** |
| **22-23** | **131 (21.7)** | **10.8 (14/130)^*^** | | **5.3 (7/131)^*^** | **2.3 (3/130)^*^** | **11.5 (15/130)^*^** | **4.6 (6/131)** | **3.8 (5/131)** | **3.5 (4/115)** | **8.6 (10/116)** |
| **24-25** | **113 (18.8)** | **9.7 (11/113)** | | **2.7 (3/113)** | **1.8 (2/113)** | **9.7 (11/113)** | **0.9 (1/113)** | **0.0 (0/113)** | **2.1 (2/95)** | **3.2 (3/95)** |
| **26-27** | **94 (15.6)** | **12.9 (12/93)^*^** | | **6.4 (6/94)** | **5.4 (5/93)^*^** | **14.0 (13/93)^*^** | **2.1 (2/94)** | **1.1 (1/94)** | **1.2 (1/82)** | **2.4 (2/83)** |
| **28-29** | **82 (13.6)** | **18.3 (15/82)** | | **2.4 (2/82)** | **1.2 (1/82)** | **18.3 (15/82)** | **2.4 (2/82)** | **0.0 (0/82)** | **2.8 (2/71)** | **4.2 (3/71)** |
| **30-31** | **80 (13.3)** | **23.8 (19/80)** | | **2.5 (2/80)** | **2.5 (2/80)** | **25.0 (20/80)** | **0.0 (0/80)** | **1.3 (1/80)** | **1.4 (1/74)** | **1.4 (1/74)** |
| **Positives** |  | **91** | | **33** | **26** | **94** | **20** | **15** | **14** | **30** |
| **Total (n)** | **603** | **601** | | **603** | **601** | **601** | **603** | **603** | **527**^†^ | **529**^† †^ |
| **P-value***^φ^* |  | **0.07** | | **0.02** | **<0.001** | **0.07** | **0.016** | **<0.001** | **0.84** | **0.019** |
| **P-trend***^φφ^* |  | 0.2 | | 0.004 | 0.003 | 0.16 | <0.001 | 0.001 | 0.2 | <0.001 |

^*^2 urine vials arrived at LUMC empty (n=601), one each in age group 22-23 and 26-27, respectively

*^**^* Defined as a positive urine CAA, microscopy or PCR

^†^ 527 women presented for CVL

^††^One participant from each of Community-A and Community-B had positive self-collected PCR specimens but did not present to clinic (n=529)

*^φ^*Test of homogeneity

*^φφ^*Score test for trend of odds

**Abbreviations:** CAA - Circulating Anodic Antigen, CVL – Cervicovaginal Lavage, PCR – Polymerase Chain Reaction for the detection of *Schistosoma* DNA
